# Time-Dependent Analysis of Human Neurophysiological Activities during an Ecological Olfactory Experience

**DOI:** 10.3390/brainsci13091242

**Published:** 2023-08-25

**Authors:** Alessia Vozzi, Ana Martinez Levy, Vincenzo Ronca, Andrea Giorgi, Silvia Ferrara, Marco Mancini, Rossella Capotorto, Patrizia Cherubino, Arianna Trettel, Fabio Babiloni, Gianluca Di Flumeri

**Affiliations:** 1 Department of Anatomical, Histological, Forensic & Orthopedic Sciences, Sapienza University of Rome, 00185 Rome, Italy; 2 BrainSigns Srl, Via Tirso, 14, 00198 Rome, Italyfabio.babiloni@uniroma1.it (F.B.); gianluca.diflumeri@uniroma1.it (G.D.F.); 3 Department of Molecular Medicine, Sapienza University of Rome, 00185 Rome, Italy; 4 Department of Computer, Control, and Management Engineering “Antonio Ruberti”, Sapienza University of Rome, 00185 Rome, Italy; 5 Department of Computer Science, Hangzhou Dianzi University, Hangzhou 310018, China

**Keywords:** EEG, EDA, olfactory stimulation, signal processing, physiological signals

## Abstract

It has been demonstrated that odors could affect humans at the psychophysiological level. Significant research has been done on odor perception and physiological mechanisms; however, this research was mainly performed in highly controlled conditions in order to highlight the perceptive phenomena and the correlated physiological responses in the time frame of milliseconds. The present study explored how human physiological activity evolves in response to different odor conditions during an ecological olfactory experience on a broader time scale (from 1 to 90 s). Two odors, vanilla and menthol, together with a control condition (blank) were employed as stimuli. Electroencephalographic (EEG) activity in four frequency bands of interest, theta, alpha, low beta, and high beta, and the electrodermal activity (EDA) of the skin conductance level and response (SCL and SCR) were investigated at five time points taken during: (i) the first ten seconds of exposure (short-term analysis) and (ii) throughout the entire exposure to each odor (90 s, long-term analysis). The results revealed significant interactions between the odor conditions and the time periods in the short-term analysis for the overall frontal activity in the theta (*p* = 0.03), alpha (*p* = 0.005), and low beta (*p* = 0.0067) bands, the frontal midline activity in the alpha (*p* = 0.015) and low beta (*p* = 0.02) bands, and the SCR component (*p* = 0.024). For the long-term effects, instead, only one EEG parameter, frontal alpha asymmetry, was significantly sensitive to the considered dimensions (*p* = 0.037). In conclusion, the present research determined the physiological response to different odor conditions, also demonstrating the sensitivity of the employed parameters in characterizing the dynamic of such response during the time. As an exploratory study, this work points out the relevance of considering the effects of continuous exposure instead of short stimulation when evaluating the human olfactory experience, providing insights for future studies in the field.

## 1. Introduction

The influence of odors at the brain level has been broadly studied in the scientific literature. Odors have shown the property of increasing memory recall, inducing relaxation, or even enhancing humans’ mental abilities. Aromatherapy is a discipline that has been used for millennia as an alternative medicine in different cultures, resulting in effective treatment of some disorders such as insomnia [1], anxiety [2], and depression [3]. In addition to these clinical applications, the effects of fragrances on the human mind and behavior is also of great interest for all those applications related to everyday life, such as cosmetics, personal care, nutrition, comfort, and psychophysical well-being [4,5]. The power of scents has always fascinated scientists, who tried to demonstrate their impact at the human psychophysiological level using the most advanced technologies for biosignal and neuroimaging analyses. Several studies conducted in the field of the functional magnetic resonance imaging (fMRI) have found that brain regions primarily involved in emotions, such as the amygdala, hypothalamus, and prefrontal cortex are activated during odor stimulation [6]. Regions generally allocated for memory and recognition such as the lateral and medial temporal cortex have also been implicated in these processes [7]. Some researchers have also pointed out a lateralization of the olfactory processes, as reported by Royet and Plailly in their review [8], in which the left hemisphere is described as related to emotions, while the right hemisphere is linked to memory. A broad range of literature has also investigated the effect of scents at the brain level using electroencephalograms (EEGs) [9]. Since its portability allows a natural fruition of odors, the methodology is particularly suitable for this application, with less invasiveness and in more realistic contexts. 

Patterns of brain activity in response to olfactory stimulation have been shown under different experimental conditions. EEG activity investigated in different frequency bands has been shown to be a sensitive measure for characterizing brain activity linked to odor perception and its consequent physiological responses. A study conducted by Diego and colleagues [10] pointed out a significant increase in frontal beta and alpha power associated with a feeling of relaxation that the inhalation of lavender essential oil induced. Similarly, a relaxing effect in response to lavender was argued by Sayorwan [11], who found an increase in theta and alpha power across all the investigated brain regions: left anterior, right anterior, right posterior, left posterior, and middle cerebral cortex. An increase in beta power in response to a specific odor, the essential oil of the Angelica gigas root, was also reported by Sowndhararajan [12], highlighting the potential ability of this fragrance to improve human language-learning abilities. 

Another aspect considered in this field is the characterization of the hedonic response to odors. Abbassi and colleagues [13] proposed a method for differentiating levels of pleasantness through a classification approach, which was shown to successfully discriminate olfactory responses using EEG features extracted in the theta, beta, and gamma bands. Based on the EEG, some fragrances were even demonstrated to improve some pathological conditions, such as relieving menopausal psychological symptoms. Moon and colleagues [14] proved that mid-life women experienced relaxing effects from the fragrances they were subjected to. These effects depended on the severity of the menopausal symptoms and were correlated with the modulation of two indices: an increase in the alpha power and a decrease in the ratio between the beta and alpha power. 

Reactions to olfactory stimuli are not limited to the central nervous system (CNS), i.e., the brain and spinal cord, as described so far. The brain’s limbic system, activated during emotional responses, is known to be also involved in homeostatic functions that regulate autonomic nervous system (ANS) activity [15]. Several studies have investigated the ANS’s response to odors as an expression of the emotions they induce [16,17]. In this context, Alaoui-Ismaïli and colleagues [18] outlined a correlation between self-reports on emotions attributed to scents and ANS responses, which were expressed as mean of six different parameters. This correlation was mainly evinced for positive emotions. Additionally, Bensafi [19] pointed out a correlation between two dimensions of pleasantness and arousal, emotions induced by olfactory stimulation, and two ANS parameters. In particular, he found a positive correlation between arousal and skin conductance amplitude and a negative correlation between pleasantness and heart rate. ANS parameters are also known to be sensitive to the stress response [20] and cognitive load [21] due to their link with the sympathetic nervous system [22]. Physiological characterization of odor stimulation might also be determined by the ability of a stimulus to relieve stress or cognitive load.

All the mentioned scientific literature provides a varied picture of the effects of odors at different levels of the human psychophysiological sphere. However, most of these studies assessed the mean reaction to an odor in the first few seconds of exposition without considering how a prolonged exposure time might affect it. The human physiological mechanisms correlated to the perception of odors are another large area of scientific research that is usually focused on the analysis of fast brain activity reactions in terms of event-related potentials (ERPs) [23,24]. Nevertheless, since ERP analysis aims to capture the instinctive and immediate reaction to external stimulations, the exposure duration in these studies was always in the milliseconds (ms) range. In real life, we are rarely exposed to single and instantaneous sensory stimuli, whether olfactory, auditory, or visual. On the contrary, in our daily life, we often interact with these stimuli over a prolonged period. Consider a scented candle in a room. Although the perception of this smell varies over time and habituation effects come into play, it is possible that our brains continue to be stimulated by it.

Thus, the research question underlying this study is precisely to investigate whether during an ecological olfactory stimulation, having a spontaneous fruition of the stimulus, there is a physiological interaction effect between the exposure duration factor and the odor to which one is exposed, and whether this interaction effect depends on the type of considered physiological activity.

In this study, two different scents, vanilla and menthol, were chosen for their clearly different connotations, being relaxing and energizing, respectively [25,26]. The participants were exposed to these odors while their brain (EEG) and electrodermal (EDA) activities were recorded. In particular, the EEG activity collected from the prefrontal cortex was analyzed using three descriptive parameters: the overall activation, the activation over the midline, and the asymmetry, over four frequency bands of interest, namely theta, alpha, low beta, and high beta. The skin conductance levels of the participations was analyzed via EDA in terms of its two components: the tonic (skin conductance level, SCL) and the phasic (skin conductance response, SCR) components. These physiological parameters were investigated in response to olfactory stimulation using the two odors conditions and a control no-odor condition (blank), while their time-domain dynamics were investigated considering the values they assumed at specific time intervals. Different from other studies on this topic, the present work did not investigate the response to the odors on average, but instead explored the evolution of physiological activity during an ecological odor exposure, expecting to find different patterns associated with the different odor conditions. Furthermore, to determine the possible effects of the exposure period, the analysis was performed both in the short- and long-term, considering the first 10 s following the beginning of the odor stimulation and a longer exposure of 90 s, respectively.

## 2. Materials and Methods

### 2.1. Experimental Sample and Design

In this study, 24 subjects were recruited as participants on a voluntary basis. All the subjects were females (25.46 ± 2.74 years old) to obtain a homogeneous population and an appropriate experimental sample size. The sample size was determined on the basis of scientific evidences in the neuroscientific and behavioral fields [27]. Before taking part in the experiment, in compliance with the General Data Protection Regulation (GDPR), (EU) 2016/679, all the participants signed an informed consent and related information sheet, in which all the data processing and privacy rights were reported. Additional informed consent was obtained from one of the participants to publish her image in an online open access publication. The study procedures were explained, and the participants had the chance to ask the experimenters any questions. The experiment was conducted following the principles outlined in the Declaration of Helsinki of 1975, as revised in 2000, and it received the approval of the Sapienza University of Rome ethical committee (nr. 2507/2020). The technologies for the EEG and EDA signal acquisition were set up on participants and then the task began. The experimental session was held in the industrial neuroscience laboratories at Sapienza University. The room in which the acquisitions were carried out was acoustically insulated from the rest of the laboratory. In addition, no other experiments were conducted on the days of the study to avoid outside interference.

**Figure 1:**
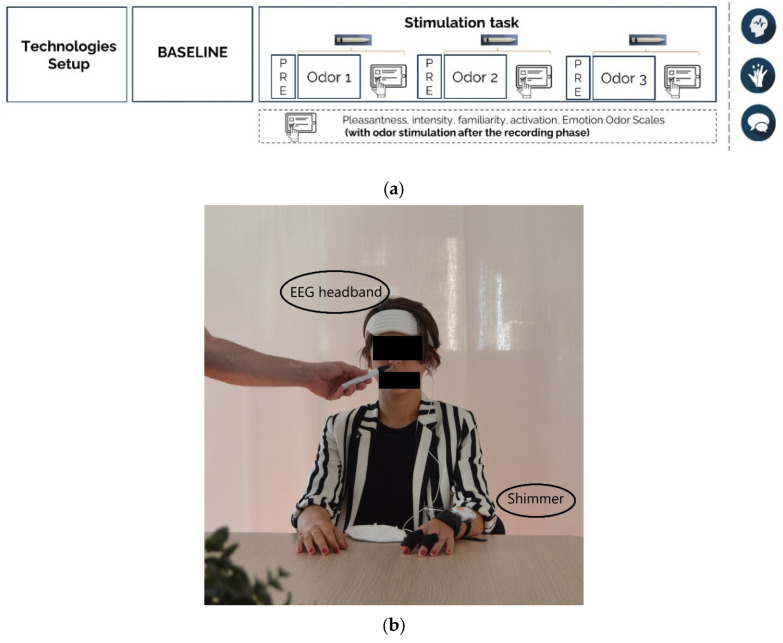
The experimental protocol. (**a**) The steps of the protocol. (**b**) Picture taken during the task.

The task lasted approximately 20 min and it consisted of two phases: the baseline and the stimulation task (Figure 1a). During the baseline phase, the participants were asked to close their eyes for 30 s and relax without any exposure to external stimuli. The stimulation task consisted of three different smelling sessions, one for each considered odor condition: vanilla, menthol, and the blank. Each session started with 30 s of pre-stimulus without stimulation, in which participants were comfortably seated in a silent room with their eyes closed in order to avoid any external interference due to other sensory channels. After the pre-stimulus, 90 s of odor stimulation occurred while the participants kept their eyes closed in silence. The stimulus was delivered through a Sniffin stick placed 3 cm from the participants’ noses (Figure 1b). The session ended with a self-assessment involving two questionnaires that were completed on a tablet while the odor was still being delivered. Between two consecutive stimulations, the room was ventilated for 3 min by opening the windows and generating forced convection motions through a fan. The odors were delivered blindly and in a randomized order for each participant. The two odors, vanilla and menthol, were assumed to be different in terms of the expected neurophysiological reactions, being related to two different psychophysiological dimensions such as relaxation and energy, respectively. This assumption was validated by the questionnaires (please refer to the following paragraph). During the blank condition, a non-odorous Sniffin stick was presented to avoid any bias, and to make participants act as if an odor was delivered.

### 2.2. Questionnaires

In the first questionnaire, the participants were asked to score the level of pleasantness, intensity, relaxation, and familiarity associated with the odor. The answers were provided using a visual analogue scale (VAS) ranging from 1 to 100, in which 1 was associated with a lower dimensional score (unpleasant, not perceived, stressful, unknown), while 100 was related to a higher dimensional score (very pleasant, very strong, relaxing, very familiar) [28]. In between the positive and negative extremes, there was a neutral label. Additionally, a questionnaire about the emotions elicited by odors was included. Two feelings, energy and soothing, were evaluated using the emotional and odor scale (EOS) [29], attempting to characterize the olfactory perception in line with the different natures of the odors. Three adjectives were reported to characterize the two feeling dimensions: (1) the energy dimension: refreshed, energetic, revitalized; and (2) the soothing dimension: relaxed, comforted, soothed. The participants were asked to indicate the intensity with which they felt these feelings, answering the question “How do you feel when you smell this perfume? Please indicate the intensity of your feeling:”. They could provide their answers using a scale from 0 (not at all) to 100 (extremely).

### 2.3. Neurophysiological Data Recording and Processing

EEG signals were recorded using a customized wearable EEG system equipped with a LiveAmp EEG (BrainProducts GmbH, Germany) amplifier [30], with eight channels placed on the forehead of the participant and two additional electrodes placed on the mastoids, which were used for the ground and reference. The electrodes were placed according to the 10–10 international system with the following channels: Fpz, Fp1, Fp2, AFz, AF3, AF4, AF7, and AF8; all referenced to the mastoids. The EEG activity was collected at a sampling rate of 250 Hz, while the impedances were kept below 20 kΩ. MATLAB software (MathWorks Inc., Natick, MA, USA) was used to analyze the raw data offline. The first step of the signal processing was to apply a notch filter at 50 Hz to remove the power-supply interference. Then, a 5th-order Butterworth bandpass filter, [2 ÷ 30] Hz, was used to remove interferences in frequencies which were not of interest. Independent component analysis (ICA) was performed using an SOBI algorithm [31] to remove the independent components related to ocular activity. Specifically, one independent component related to ocular blinks was manually selected after a visual inspection performed by the same operator: a biomedical engineer with more than 5 years of experience in EEG data analysis. The reconstructed EEG signal was segmented into epochs of 1 s in order to maintain stationarity conditions for the EEG signal [32], with 0.5 s of overlap to avoid the “boundary effect”. Three additional criteria for detecting artifacts based on the amplitude, sample-to-sample difference, and trend [33,34] of the signal were applied. The epochs identified as artifacts were removed from the dataset. The signal acquired during the baseline condition, in which participants had their eyes closed in a resting state, was employed for calculating the individual alpha frequency (IAF). According to Klimesch [35], the IAF is used to define the individual EEG frequency bands of each participant. In the present study, four EEG frequency bands were considered: theta [IAF − 6 Hz, IAF − 2 Hz], alpha [IAF − 2 Hz, IAF + 2 Hz], low beta [IAF + 2 Hz, IAF + 10 Hz], and high beta [IAF + 10 Hz, IAF + 18 Hz]. The EEG signals were filtered into these specific sub-bands, and the power in the time domain tP (1) was computed across each electrode for each signal $x\left(n\right)$ with a period $N_{0}$.

(1) $$tP=\frac{1}{N_{0}}\sum_{n=0}^{N_{0}-1}{\left|x\left(n\right)\right|}^{2}$$

The tP filtered in b band over the ch channel was normalized with respect to the baseline as in (2):

(2) $$norm-tP_{b,ch}=\frac{stimtask-tP_{b,ch}-median(bas-tP_{b,ch})}{MAD\left(bas-tP_{b,ch}\right)}$$

where $stimtask-tP_{b,ch}$ and $bas-tP_{b,ch}$ are the signal powers *P* computed during the stimulation task and during the baseline, respectively, while *median*$\left(bas-tP_{b,ch}\right)$ and $MAD\left(bas-tP_{b,ch}\right)$ are the median and the median absolute deviation of $bas-tP_{b,ch}$. In other words, this is a normalization similar to the z-score, but because it uses the median-related parameters instead of the mean, it is known as robust normalization [36]. In order to resume the synchronous de-/activation of brain cortical regions of interest, the global field power (GFP) was derived from tP over the N electrodes of interest [37,38], as described by the following Formula (3):

(3) $$GFP_{b,N}=\frac{1}{N}\sum_{i=1}^{N}tP_{b,N}$$

$tP_{b,N}$ in this case, is intended as the normalized tP.

For each frequency band b, three different clusters of electrodes were considered. For each of them, the GFP was computed, then three synthetic descriptive indices were computed as follows, including the overall frontal GFP (OaGFP) (4), the frontal midline GFP (FmGFP) (5), and the GFP asymmetry (AsGFP) (6):

(4) $$Oa-GFP_{b}=GFP_{b,ALL},\;\mathrm{where}\;\mathrm{ALL}=\mathrm{Fpz},\;\mathrm{Fp1},\;\mathrm{Fp2},\;\mathrm{AFz},\;\mathrm{AF3},\;\mathrm{AF4},\;\mathrm{AF7},\;\mathrm{AF8}$$

(5) $$Fm-GFP_{b}=GFP_{b,C},\;\mathrm{where}\;C=\mathrm{Fpz},\;\mathrm{AFz}$$

(6) $$As-GFP_{b}=GFP_{b,R}-GFP_{b,L},\;\mathrm{where}\;R=\mathrm{AF8},\;\mathrm{AF4},\;\mathrm{and}\;L=\mathrm{AF7},\;\mathrm{AF3}.$$

where ALL refers to all the frontal electrodes, C refers to the central midline electrodes, R refers to the right frontal electrodes, and L refers to the left frontal electrodes.

The EDA was recorded using a Shimmer3 GSR+ unit device (Shimmer Sensing, Dublin, Ireland). Two sensors were placed on the palmar side of the proximal phalanx of the second and third fingers of the non-dominant hand of the participants. The signals were acquired at a sampling frequency of 64 Hz. The EDA was low-pass filtered using a cut-off frequency of 1 Hz, then processed through the Ledalab suite: an open-source toolbox in MATLAB (MathWorks, Natik, MA, USA) for EDA processing. A continuous decomposition analysis [39] was applied to estimate the tonic (SCL) and phasic (SCR) components [40]. The SCL is the slow-changing component of the EDA signal, while the SCR is the fast-changing component, and it is usually related to single-stimulus reactions. The EDA components were estimated using a 2 s time resolution. Each value assumed by the SCL and SCR in the considered time period was normalized according to the method proposed by Lykken and colleagues [41,42] as follows:

(7) $$SCL_{norm}=\frac{taskSCL-\min\left(taskSCL\right)}{\max\left(taskSCL\right)-\min\left(taskSCL\right)}$$

(8) $$SCR_{norm}=\frac{taskSCR}{\max\left(taskSCR\right)}$$

where taskSCR and taskSCL are the values of SCL and SCR measured during the entire individual experimental session, i.e., during the baseline and stimuli.

### 2.4. Statistical Analysis

Statistical analyses were performed on the questionnaires and neurophysiological measures. In both cases, the Shapiro–Wilk test of normality [43] demonstrated that the data were Gaussian; hence, parametric tests were employed in the analyses.

In terms of the questionnaires, the statistical analysis was initially performed on the answers regarding pleasantness, intensity, relaxation, and familiarity. In this case, four ANOVAs (analysis of variance) [44], one for each dimension, were conducted, considering the different odors as the factors. The Duncan post-hoc test was performed to assess differences among the values assumed in the three conditions. Concerning the emotional scale, a radar graphic was initially reproduced to screen for the dimension for which a difference in the conditions was expected. Over each selected dimension, an ANOVA was performed to validate the significance of the differences among the three odor conditions.

Then, statistics on the EEG and EDA indices were performed to assess their temporal evolution in accordance with the odor presentation. It is worth noting that three subjects were excluded from both the EEG and EDA analysis due to recording issues, while an additional subject was excluded only from the EEG analysis for the same concerns.

For each odor condition, two different analyses were performed to assess the short-term and long-term neurophysiological responses to the stimuli. For the short-term response, only the first 10 s of odor exposition were investigated, averaging data with a time resolution of 2 s. Five time intervals were considered in this analysis: from 1 s to 2 s (1–2), from 3 s to 4 s (3–4), from 5 s to 6 s (5–6), from 7 s to 8 s (7–8), and from 9 s to 10 s (9–10). For the long-term response, the entire odor stimulation was explored, and the data were averaged with a time resolution of 10 s. In this analysis, five representative time intervals were selected: at the beginning of the odor delivery (1–10), from 11 s to 20 s (11–20), from 21 s to 30 s (21–30), from 51 s to 60 s (51–60), and from 81 s to 90 s (81–90). These intervals were chosen to identify five segments of interest in order not to lose statistical power due to the limited sample size. In this regard, the approach was to investigate the first 30 s, then after one minute and after one minute and a half.

Furthermore, the data were averaged during the pre-stimulus session. An initial qualitative analysis was performed through a visual inspection of the headplots generated for each frequency band, each kind of stimulation, and each odor condition. Then, a statistical analysis was performed on the neurophysiological variables using 3 × 5 ANOVAs, one for each variable, considering the three odor conditions and the five temporal segments as factors. This analysis was conducted for both the short-term and long-term responses without including the pre-stimulus condition. For the purpose of this study, only the interactions between the odor conditions and the time segments were reported as results, since the experiment was designed to differentiate the physiological responses to different odor stimuli at different time points from the beginning of the stimulation. The main effect of the fragrance and the main effect of the time were not counted in the results since they were not informative in this study. Indeed, the first effect does not consider the time evolution of the response, which is a factor of interest for the present study, while the second does not consider the heterogeneity of the stimuli. The pre-stimulus was considered for assessing whether the differences between the sessions were effectively correlated to the odor stimulation or whether they may have been biased from the beginning. For each index and each band, an ANOVA was performed over the pre-stimulus data for the three odor conditions.

## 3. Results

The results have been organized into three different subsections, questionnaires, EEG topographical analyses, and neurophysiological parameters, in order to facilitate the reader. All the results are briefly described, pointing out the relevant findings. The results will be commented on more comprehensively in the discussion section.

### 3.1. Questionnaires

**Figure 2:**
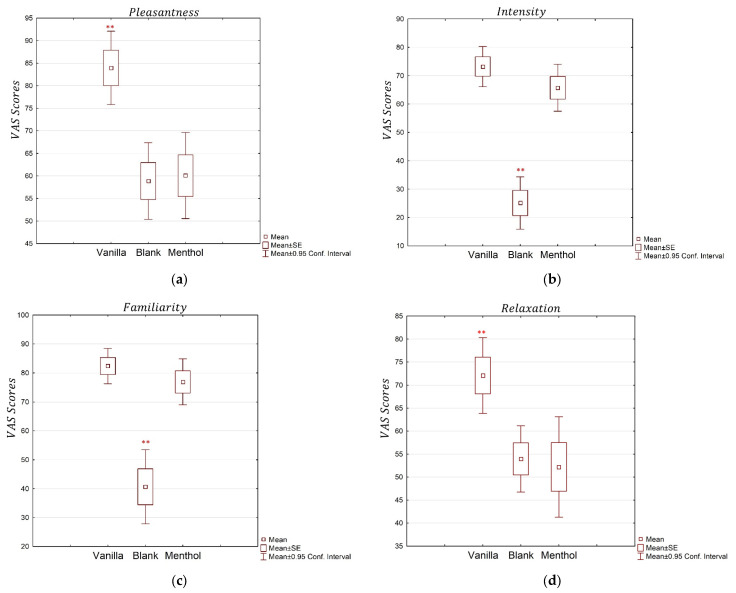
The average VAS scores for the four explored dimensions: pleasantness (**a**), intensity (**b**), familiarity (**c**), and relaxation, and (**d**) for the three odor conditions: vanilla, blank, and menthol. “**” indicates when the VAS values are significantly different from the others, with *p* < 0.01.

The ANOVAs performed on the four VAS values associated with the questions about the odor pleasantness, intensity, relaxation, and familiarity showed a significant effect of the type of odor delivered as the stimulus (Figure 2). The results showed a significant effect of the odors for all the dimensions. In particular, the ANOVA for the pleasantness dimension achieved a significant effect (partial *η*^2^ = 0.32, *p* = 0.0001), and the post-hoc test revealed significantly higher scores for the vanilla compared to the menthol and the blank. The ANOVA for the intensity dimension revealed a significant effect of the odors (partial *η*^2^ = 0.718, *p* < 0.0001), with lower scores for the blank odor compared with the menthol and vanilla. The familiarity dimension was also significantly different in the three conditions (partial *η*^2^ = 0.530, *p* < 0.0001), with lower scores for the blank stimulation compared to the others. The relaxation dimension showed a significant result (partial *η*^2^ = 0.207, *p* = 0.0048), with higher scores for the vanilla when compared to the menthol and blank.

**Figure 3:**
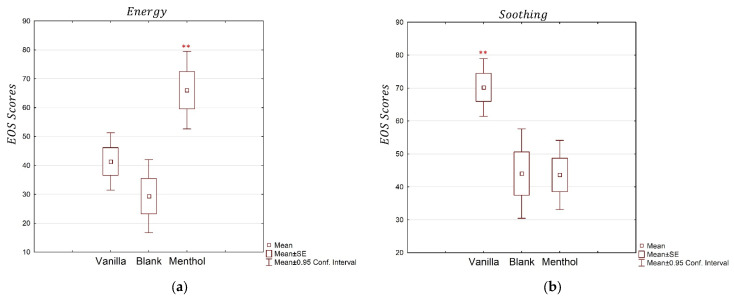
The average EOS scores for the two considered emotions, the energy (**a**) and soothing (**b**) dimensions, for the three odor conditions: vanilla, menthol, and blank. “**” indicates when the VAS values are significantly different from the others, with *p* < 0.01.

The analysis of the energy and soothing dimensions based on the emotion and odor scale showed a significant effect of the odors for both the investigated emotions (Figure 3). The energy dimension revealed a significant effect of the stimulus (partial *η*^2^ = 0.374, *p* < 0.0001). Higher scores were observed for the menthol compared to the blank odor and the vanilla. Additionally, the soothing dimension showed a significant effect (partial *η*^2^ = 0.280, *p* = 0.0005), with higher scores for the vanilla odor than for the blank and the menthol, supporting the working assumption regarding the different natures of the two selected odors.

### 3.2. EEG Topographical Analysis

Concerning the EEG data analysis, below are the headplots representing the signal power distribution for each odor condition and each considered time-segment across all frequency bands of interest for both short- and long-term responses, averaged over the whole sample. This representation aims to provide a topographical overview of brain rhythm time-domain dynamics over the whole investigated area. In the following paragraphs, a more detailed analysis, including statistics, will be provided for the previously described synthetic indicators.

From a qualitative point of view, activation patterns over different clusters of electrodes are remarkable in different frequency bands depending on the kind of odor. It is noteworthy that all the values are normalized with respect to the baseline (please refer to Section 2.3), i.e., the 0 value in the figures.

**Figure 4:**
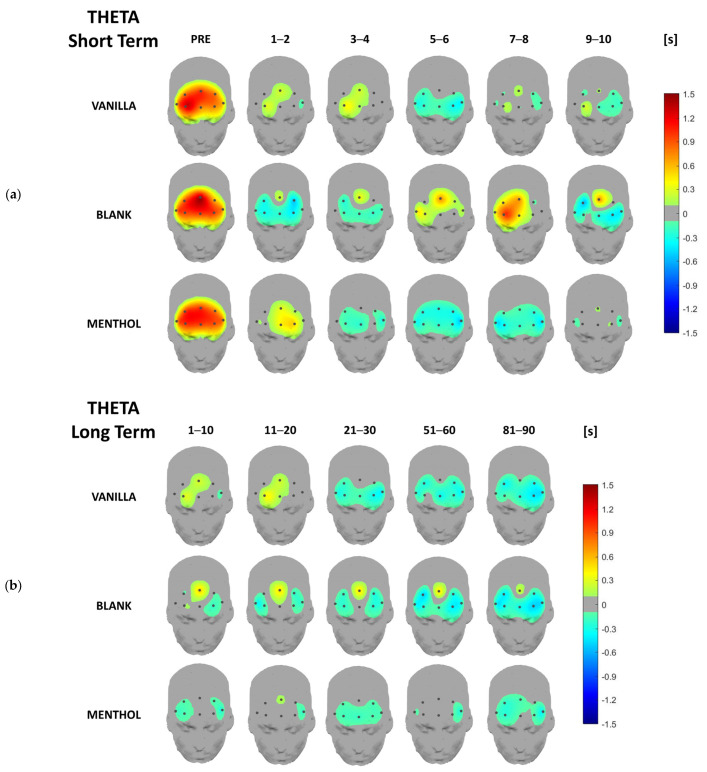
Headplots representing the theta power distribution in the short-term (**a**) and long-term (**b**), averaged over the whole sample for the three stimulations and the different time segments.

The headplots in the theta band (Figure 4) showed that the olfactory stimulation induced an overall power suppression, i.e., a desynchronization with respect to the high synchronization characterizing the pre-stimulus phase over all the considered sites. On a short-term scale, it is interesting to note that while the vanilla and menthol theta rhythms fully decreased (green–blue color indicates negative values) after 4 and 2 s with the bank stimulus, there was a new theta increasing over the central sites between 6 and 8 s, respectively. Furthermore, it is remarkable that to a lesser extent, in the blank odor condition, the theta power over the central sites exhibited positive values during the blank condition, even on the long-term time scale until the end. On the other hand, the theta power increased centrally until 20 s with the vanilla, while with the menthol, no synchronization phenomena were found.

**Figure 5:**
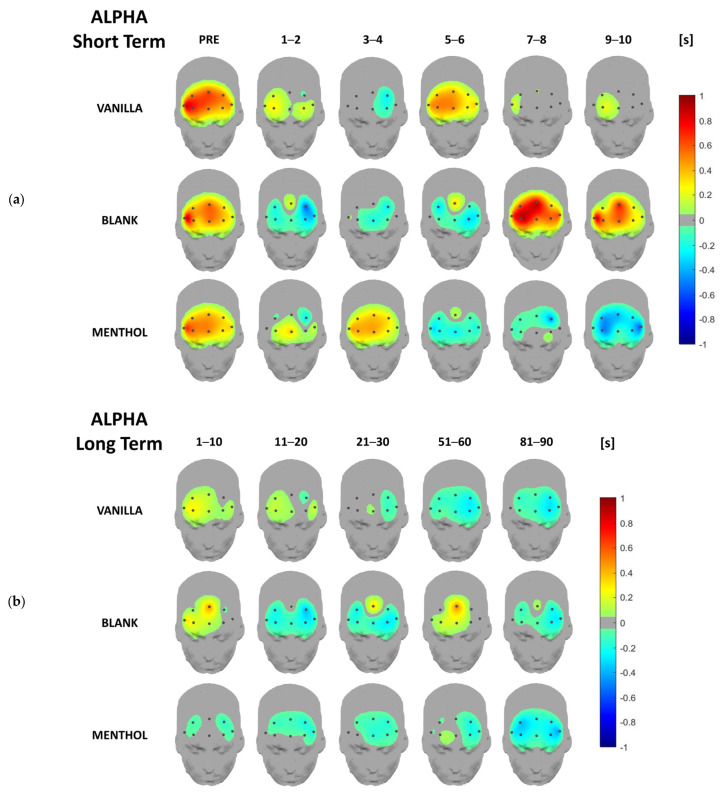
Headplots representing the alpha power distribution in the short-term (**a**) and long-term (**b**), averaged over the whole sample for the three stimulations and the different time segments.

Additionally, the alpha power was modulated by the kind of odor. On a short-term scale, the headplots show a remarkable power increasing for the blank condition which occurs at 7–8 s from the odor exposition, even achieving similar pre-stimulus levels after initially decreasing in the first seconds (Figure 5a). The vanilla showed a similar effect, i.e., an initial decrease followed by an increase in new alpha power, but over a shorter time (between 5 and 6 s). On the contrary, the menthol appeared to generate a power decrease within the first four seconds after the odor exposition.

In the long-term, the alpha power for both the odors seemed to decrease overall, showing a slight asymmetry towards the left hemisphere, while a new alpha increase over the central sites was measured with the blank odor after 1 min (Figure 5b).

**Figure 6:**
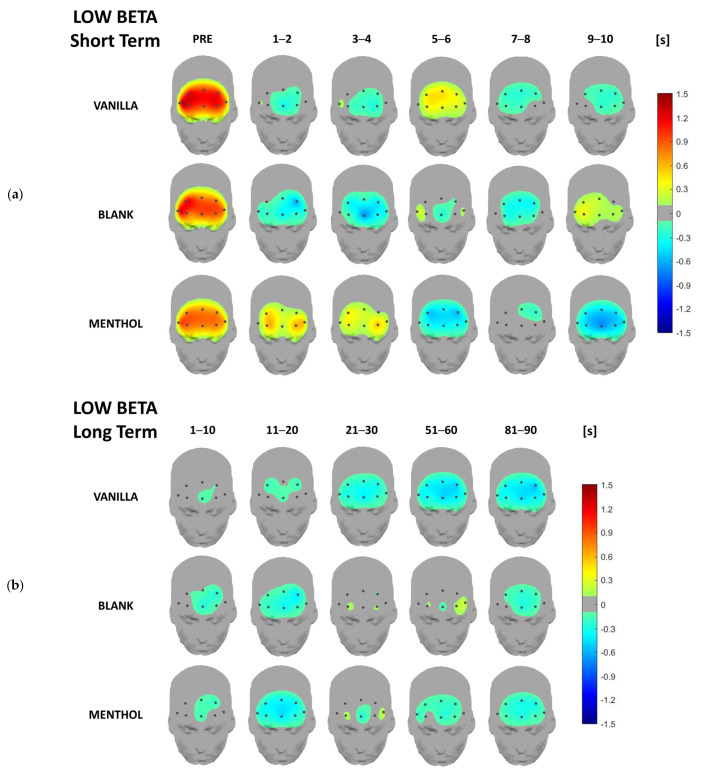
Headplots representing the low beta power distribution in the short-term (**a**) and long-term (**b**), averaged over the whole sample for the three stimulations and the different time segments.

In the short-term, the vanilla caused an immediate decrease in low-beta rhythms apart from a few and short increase after 5 s. The blank odor caused a similar trend, but with a new increase at the end of the ten seconds. On the contrary, the menthol did not cause a fast reaction, but instead a more “linear” trend, with a fully synchronized decrease over the whole prefrontal cortex at the tenth second (Figure 6a). On a long-term scale, no specific patterns were found (Figure 6b).

**Figure 7:**
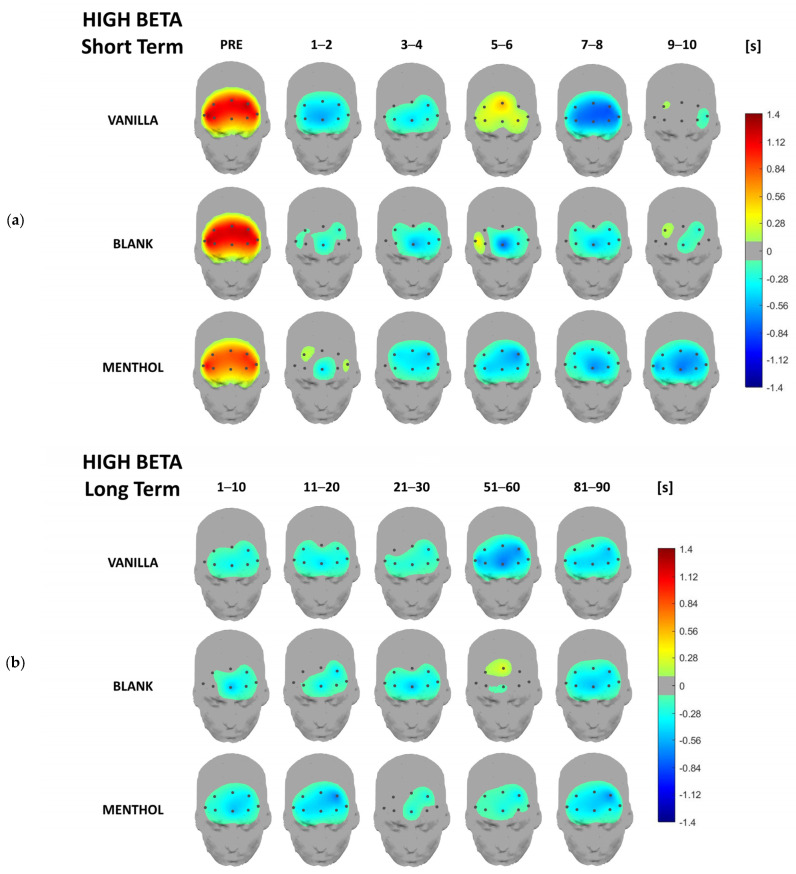
Headplots representing the high-beta power distribution in the short-term (**a**) and long-term (**b**) averaged over the whole sample for the three stimulations and the different time segments.

The most remarkable pattern in the high beta band was associated with the power increasing between 5 and 6 s in the short-term (Figure 7a), especially over the central sites for the vanilla stimulation. On the other hand, menthol caused a linear and synchronized activity that decreased over the entire prefrontal cortex.

On a long-term scale, no specific patterns were found (Figure 7b). Both the odors caused an overall de-activation, i.e., a power decrease, throughout the 90 s, while the blank odor did not cause such a clear trend, as a short increase was measured after 1 min.

### 3.3. Neurophysiological Parameters

An analysis of the synthetic indices was conducted to identify any particular EEG patterns that were eventually significant among those observed in the headplots. An ANOVA performed over the pre-stimulus time frame showed that there was no difference among the three odors for any of the considered indices (*p* >> 0.05), i.e., not surprisingly, the brain activation was similar to the condition when waiting for the stimulation. Instead, during the odor stimulation, several interactions between the odor condition and the considered time frames identified through the ANOVAs, and in this section, they are reported according to (i) the frequency bands of interest, i.e., theta, alpha, low beta, and high beta, and (ii) the type of response, i.e., the short- and long-term response.

**Figure 8:**
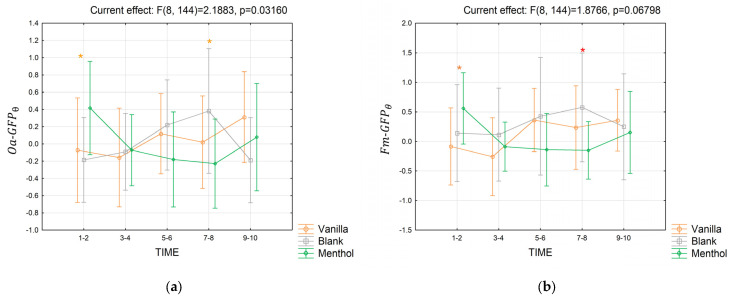
The trends in the $Oa-GFP_{\theta }$ (**a**) and $Fm-GFP_{\theta }$ (**b**) indices for the three odor conditions in the short-term. Vertical bars denote 0.95 confidence intervals. Red “*” indicates when the index for a considered time segment is significantly different for an odor condition compared to the others, with *p ≤* 0.05. “*” is colored in orange when 0.05 *< p ≤* 0.09.

In the theta band, considering the short-term response, the analysis performed over $Oa-GFP_{\theta }$ values (Figure 8a) showed a significant interaction between the odor stimulation and the time segments (partial *η*^2^ = 0.11, *p* = 0.03). A Duncan post-hoc test showed that during the first two seconds (1–2), the index for the blank stimulation antly lower on average than for the menthol (*p* = 0.06), while at 7–8 s, the effect was opposite and the index assumed higher values for the blank compared to the menthol (*p* = 0.05). Still, in the short-term analysis, the interaction for $Fm-GFP_{\theta }$ (Figure 8b) was not strictly significant, but it was closed to the threshold (partial *η*^2^ = 0.09, *p* = 0.068). Considering only the central electrodes, the difference between the theta GFP during the menthol and vanilla stimulation was enhanced during the first 2 s (1–2) (*p* = 0.057), while in 7–8 s, the same index decreased for the menthol stimulation compared to the blank (*p* = 0.035). No interactions were found for the $As-GFP_{\theta }$. No significant results were encountered in the long-term analysis.

**Figure 9:**
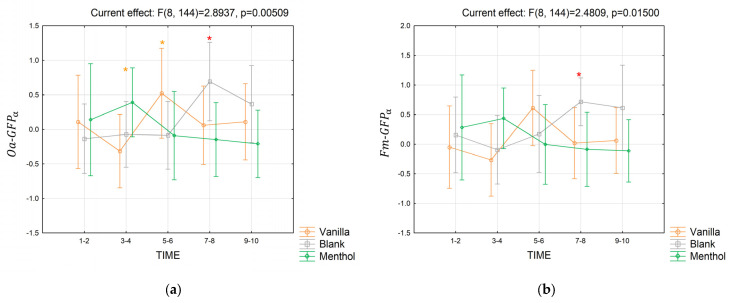
The trend in the $Oa-GFP_{\alpha }$ (**a**) and $Fm-GFP_{\alpha }$ (**b**) indices for the three odor conditions in the short-term. Vertical bars denote 0.95 confidence intervals. Red “*” indicates when the index for a considered time segment is significantly different for an odor condition compared to the others, with *p ≤* 0.05. “*” is colored in orange when 0.05 *< p ≤* 0.09.

Considering the alpha frequency band, both $Oa-GFP_{\alpha }$ (Figure 9a) and $Fm-GFP_{\alpha }$ (Figure 9b) were significantly modulated by the odor condition and the considered time segments for the short-term response. Indeed, a significant interaction was found for $Oa-GFP_{\alpha }$ (Partial *η*^2^ = 0.138; *p* = 0.005), pointing out that: in seconds 3–4, the index tended to assume higher values for the menthol compared to the vanilla stimulation (*p* = 0.053); in seconds 5–6, the index tended to be significantly higher during the vanilla stimulation compared to the others (*p* = 0.09 for both); in seconds 7–8, the index for the blank condition was significantly higher than for menthol (*p* = 0.01), while it tended to be higher than vanilla (*p* = 0.07). Also, the interaction for $Fm-GFP_{\alpha }$ was significant (partial *η*^2^ = 0.12, *p* = 0.015), and the Duncan post-hoc analysis revealed that in seconds 7–8, the blank condition elicited a higher index compared to vanilla (*p* = 0.066) and menthol (*p* = 0.03).

**Figure 10:**
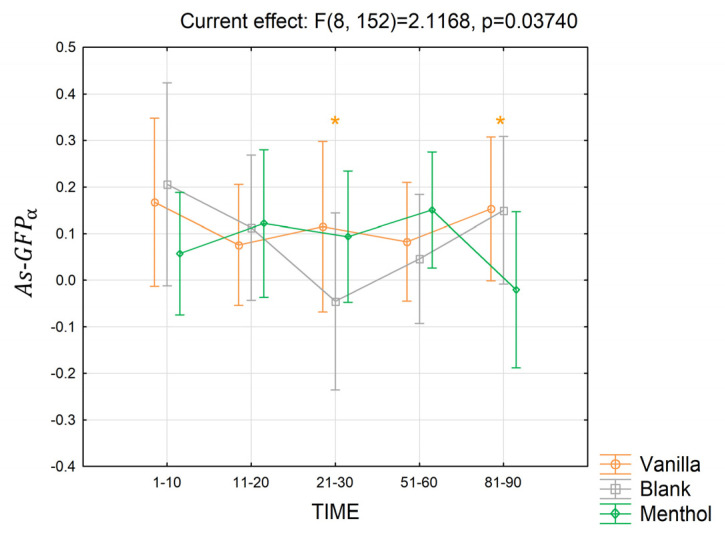
The trend in the $As-GFP_{\alpha }$ for the three odor conditions in the long-term. Vertical bars denote 0.95 confidence intervals. “*” is colored in orange when 0.05 *< p ≤* 0.09.

Also, the asymmetry, i.e., the $As-GFP_{\alpha }$ (Figure 10) index, was significantly modulated by the fragrances and the time, but only considering the long-term response (partial *η*^2^ = 0.10, *p* = 0.037). The index was lower, on average, in the blank condition compared to the menthol (*p* = 0.07) and the vanilla (*p* = 0.07) in seconds 21–30, while it decreased for menthol compared to both the vanilla and blank (*p* = 0.06, *p* = 0.06) in seconds 81–90. No other significant interactions were found in the alpha band.

**Figure 11:**
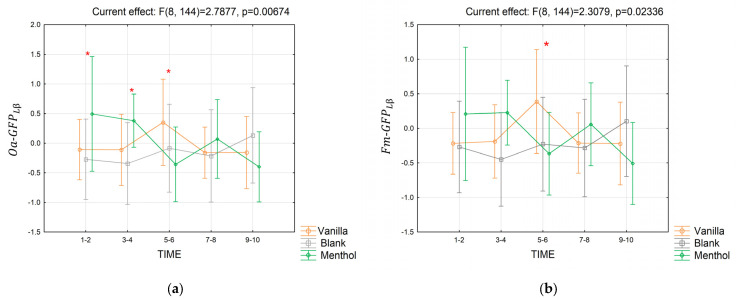
The trend in the $Oa-GFP_{L\beta }$ (**a**) and $Fm-GFP_{L\beta }$ (**b**) indices for the three odor conditions in the short-term. Vertical bars denote 0.95 confidence intervals. Red “*” indicates when the index for a considered time segment is significantly different for an odor condition compared to the others, with *p ≤* 0.05.

Concerning the low beta band, significant interactions for both $Oa-GFP_{\beta L}\;$(Figure 11a) and $Fm-GFP_{L\beta }$ (Figure 11b) were found in the short-term response (partial *η*^2^ = 0.13, *p* = 0.0067 and partial *η*^2^ = 0.11, *p* = 0.02). The Duncan analysis revealed that in seconds 1–2, $Oa-GFP_{L\beta }$ assumed higher values during the menthol stimulation compared to the vanilla and blank (*p* = 0.07 and *p* = 0.027), while in seconds 3–4, it was higher for the menthol compared to the blank (*p* = 0.038). In seconds 5–6, it was higher for the vanilla compared to the menthol (*p* = 0.04). $Fm-GFP_{L\beta }$ assumed higher values in seconds 5–6 during the vanilla stimulation compared to the blank (*p* = 0.09) and menthol (*p* = 0.04). No effect was found for the long-term response.

**Figure 12:**
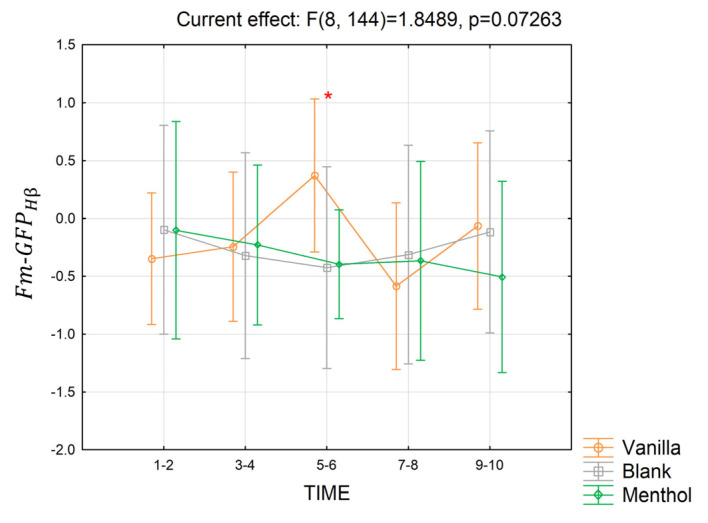
The trend in the $Fm-GFP_{H\beta }$ index for the three odor conditions in the short-term. Vertical bars denote 0.95 confidence intervals. Red “*” indicates when the index for a considered time segment is significantly different for an odor condition compared to the others, with *p ≤* 0.05.

In the high beta band, only the interaction for $Fm-GFP_{H\beta }$, even if not strictly significant, had a *p*-value closed to the threshold of significance (partial *η*^2^ = 0.09, *p* = 0.07) for the short-term response (Figure 12). The post-hoc test showed that in seconds 5–6, the index was higher for vanilla compared to the blank and menthol (*p* = 0.014 and *p* = 0.018).

**Figure 13:**
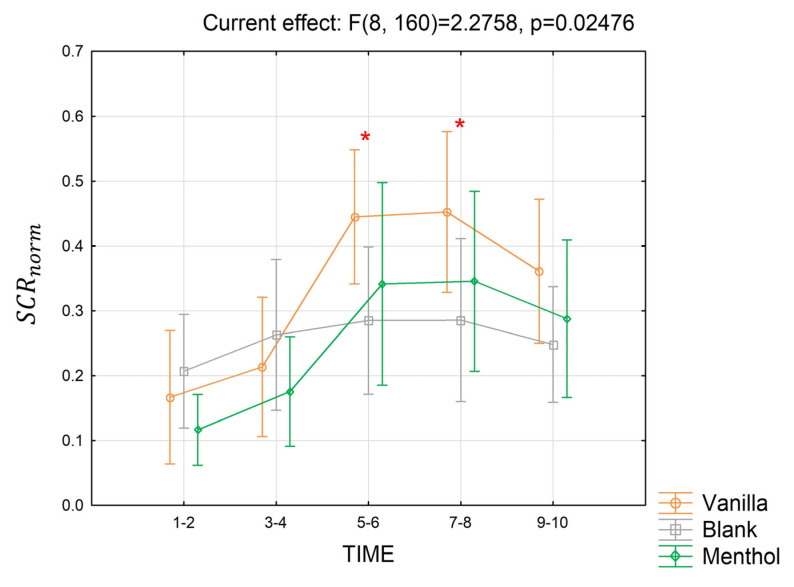
The trend in the $SCR_{norm}$ index for the three odor conditions in the short-term. Vertical bars denote 0.95 confidence intervals. Red “*” indicates when the index for a considered time segment is significantly different for an odor condition compared to the others, with *p ≤* 0.05.

The analysis of the EDA parameters did not provide any significant effect in terms of SCL in the short- and long-terms or in terms of the long-term SCR, while it showed a significant interaction between odor x time segments over the $SCR_{norm}$ (Figure 13) values for the short-term response (partial *η*^2^ = 0.10, *p* = 0.025). The Duncan post-hoc analysis revealed that in seconds 5–6 and 7–8, the $SCR_{norm}$ for the vanilla stimulation increased, becoming significantly higher than the blank (*p* = 0.006 and *p* = 0.004), and had a tendency towards menthol (*p* = 0.07 and *p* = 0.06).

All the results have been summarized in the following table (Table 1), providing an overview of whether a significant (green flag) or almost significant (orange flag) effect was obtained.

## 4. Discussion

The questionnaire results regarding familiarity, intensity, relaxation, and pleasantness showed differences in the explicit level of odor perception. These data confirmed that at the explicit level, the odors were differently perceived and discriminable. The answers to the emotion scale also pointed out that the odors were characterized as expected by the participants. Indeed, they declared feeling more relaxed, comforted, and soothed while perceiving the vanilla, and more refreshed, energetic, and revitalized for the menthol stimulation. This is in line with the labels attributed to the odors in the phase of the experimental design: relaxing for vanilla and energizing for menthol. The blank odor, not surprisingly, induced low levels of emotions compared to the others. This preliminary analysis validated the experimental hypothesis about the stimuli, supporting the working assumption that the physiological effects were not due to chance, but instead because of a different sensorial experience based on two opposite psychosomatic dimensions, i.e., energy and relaxation.

The analysis of the neurophysiological indices, together with the headplot qualitative analysis, succeeded in characterizing the temporal evolution of the indices, especially in the short-term, for the different EEG frequency bands of interest.

The headplots in the theta band showed that unlike the other conditions, the blank condition entailed a central power increase at 7–8 s. This was confirmed by the significant interaction found at the level of the global theta activity and at the central sites, with an increase in the indices for the blank compared to the menthol. The frontal GFP in the theta band is known to be associated with the mental effort required by participants while accomplishing a task, as supported by numerous scientific studies [45,46,47,48]. These findings, together with the initial theta GFP increasing in the first two seconds of menthol exposure, might be related to phenomenon of odor recognition which occurred when the participants smelled the fragrances. During the first two seconds, the participants easily recognized the menthol, also judged as familiar in the self-assessments, entailing an increase in workload that might be related to the first reaction to the well-known odor. After the first two seconds, i.e., after the recognition, the frontal midline index decreased for the menthol stimulation, moving from being higher than the blank to being significantly lower. These findings are in line with the study by Skorić and colleagues [49], who pointed out a theta reduction over central brain regions in response to the odors of peppermint and lemon. Also, Martin [50] found a significant decrease in the theta activity associated with the most pleasant odor, arguing this was due to a reduced level of attention and cognitive load during the olfactory perception. The blank condition instead did not generate a significant initial response, but it caused a plausible increase in the workload after some seconds from the beginning of the exposition, likely due to the effort that the participants experienced in trying to recognize an odor that did not occur.

In the short-term, also alpha band also allowed for a discrimination between the different brain responses to the three odor conditions during this time. The first difference that can be pointed out, as occurred in theta band, is the discrimination between the odor and no-odor stimulations. The blank generated a brain reaction until 7–8 s in which the GFP significantly increased, achieving a value significantly higher compared to menthol and vanilla. Alpha rhythms are usually linked to rest and inhibition of cognitive processes and sensorial processing [51], while it has been demonstrated to desynchronize, i.e., to reduce its power, as a reaction to external stimulations and events [52]. An increase in alpha power can be reconducted to synchronization phenomena, which occur in the absence of external stimulations. This was the case of the blank stimulus, in which the participants began the experiment expecting to smell an odor and the odor did not occur. The analysis of the alpha activity also highlights a different response to the two fragrances. As shown by the headplots, menthol and vanilla induced opposite trends in 3–4 and 5–6 s. The general frontal alpha increase found for vanilla moving from 3–4 to 5–6 s might imply a cue of relaxation, which occurs after some seconds after exposure to the scent [53].

The sole effect in the long-term was found for the alpha asymmetry, which was generally linked to the approach–withdrawal behavior and to motivational attitudes [38,54]. In general, positive asymmetries are linked to approach behavior, while negative values are linked to adverse mood, i.e., withdrawal. In this case, the alpha asymmetry decreased for the blank in 21–30 s, and this might reflect the frustration that the participants felt when trying to recognize the odor for a long time without succeeding. On the other hand, both the odors caused positive asymmetries throughout the entire exposure, i.e., a pleasant effect due to the presence of the scents. A significant decrease was found for menthol in the last 10 s; an effect that could be linked to a sort of discomfort caused by an intense fragrance if smelt for prolonged time.

The analysis of the beta band was informative mainly in the low beta, for which the interaction between the odor conditions and time was significant at both global and central leads. Although the overall low beta activity had a mild modification for the blank condition, it was significantly modulated by exposure to the fragrances. As was also observed at the level of the headplots, during the first three considered time segments, the brain activity in the low beta band initially assumed low values, then increased for the vanilla. Meanwhile, it went in the opposite direction for the menthol stimulation, during which it initially assumed high values, then decreased. Beta rhythms, especially over the prefrontal cortex, are known in the literature to be linked to the high cognitive functions, decision-making, and alert states [55,56]. In the context of olfactory perception, beta modulation was associated with the relaxing property of the scents, which were assumed to decrease the participants’ levels of alertness [57]. In some studies, beta activity was even associated with a hedonic response to fragrances. This is the case for the study conducted by Sayowan and colleagues [58], which found an anterior beta increase that was associated with the smelling of jasmine oil. This was evaluated as elicitor of positive emotions and feeling of well-being. Additionally, Watanuki and Kim [59] found a left beta activation in response to a pleasant odor.

The analysis of the ANS parameters revealed a significant effect of the odors in the modifications of the SCR, which significantly increased after some seconds of vanilla stimulation. In the literature, a SCR increase has been associated with an increase in the emotional arousal induced during fragrance perception [19]. This was also found in other contexts. Winter and colleagues [21], among others, demonstrated an increase in arousal associated with a higher cognitive load, followed by an SCR increase in response to comprehension tasks. Not surprisingly, this effect was not found for the SCL parameter, which is known to respond with a certain latency to external stimulations.

In general, almost all the effects pointed out in this study were associated with the short-term response. As reported in the review edited by Lorig [60], the reaction times to odors is very subjective, and it also changes at the level of a single subject according to the property of the odor itself. Nevertheless, it is reasonable to assume that the reaction to the fragrances occurred in the first seconds of the odor exposition, where the interactions emerged. In the long-term, we hypothesize a habituation effect [61] for which the brain activity is no longer influenced by the kind of stimulus, but intead by the task itself.

Despite the promising outcomes of this study, some limitations must be discussed. Initially, in line with the article published by the authors [27], the sample size could be increased in order to improve the reliability of the obtained results. A larger sample size would also allow us to consider a higher time resolution as a factor in the analysis. Furthermore, the methodology employed in the study might be reproduced with a higher number of stimuli in order to associate the obtained results to the energizing or relaxing properties of the odors rather than to the type of odor employed as stimulus. Another stimulus-related limitation is that menthol, unlike vanilla, has a strong trigeminal component. It might therefore be useful to include odors that have the same type of component among the stimuli for a more controlled comparison. An additional study might also involve a gender-balanced population, increasing the number of participants to generalize the findings of this work.

Of course, the provided discussions must be intended not as definitive evidence, but rather as deductions made on the basis of the concomitant analysis of the obtained results and the previous scientific literature. Regardless, this work demonstrated that it is crucial to take into account the time-domain dynamics of the physiological variables because the average over fixed time frames, such as over the entire 10 s, could provide relevant information related to the users’ psycho-behavioral reactions.

## 5. Conclusions

The present study aimed to investigate how human physiological activities evolve in response to different odors. The participants experienced three different odor conditions, vanilla, menthol, and a blank, while their EEG and EDA signals were analyzed in the short- and long-term time scales. The results demonstrated significant interactions between the odor conditions and the time frames for Oa−GFP in the theta (*p* = 0.03), alpha (*p* = 0.005), and low beta (*p* = 0.0067) bands, and for the Fm−GFP in the alpha (*p* = 0.015) and low beta (*p* = 0.02) bands in the short-term analysis. Additionally, the $SCR_{norm}$ was significantly modulated by the time variable in the-short term, depending on the considered odor condition (*p* = 0.024). In terms of the long-term effects, instead, only one EEG parameter, the $As-GFP_{\alpha }$, significantly discriminated the odour conditions at the different time points (*p* = 0.037). Through a purely deductive exercise, the frontal theta activity was linked to the mental effort allocated to recognizing the odors; in fact, it only increased over time in the blank condition. Alpha suppression was linked to reactive phenomena to external stimuli. The alpha rhythms did not decrease in blank condition as they did with the two odors. Additionally, the alpha asymmetry, usually linked to pleasantness, demonstrated a sort of discomfort when exposed for prolonged times to menthol, while it achieved higher values with exposure to vanilla. The beta rhythms, linked in previous studies to the hedonic dimension, showed a higher increase with exposure to vanilla. Finally, the fast components, i.e., the SCR of skin sweating that is usually linked to physiological arousal, highlighted a fast effect caused by the odors (vs. the blank condition) that disappeared in the long-term.

In conclusion, the present research demonstrated not only a measurable and discriminable physiological response to different olfactory stimulation, but also the sensitivity of each physiological parameter and therefore their dynamics over time. In addition to this inferred insight, this exploratory study demonstrates the relevance of considering the effects of exposure at different time points instead of averaging the overall response to the stimulation, providing insights for future research aiming to characterize the human olfactory experience in any field. Since the effect of the odors might be different at different time points from the beginning of the exposition, it is important to take the effect of time into account to describe the effects associated with odor exposure.

## Figures and Tables

**Table 1:** Summary of the significant results for the considered physiological parameters in the short- and long-term. The significant effects (*p ≤* 0.05) are reported with a green flag, while the almost significant effects 0.05 *< p ≤* 0.09 are marked with an orange flag.

		SHORT-TERM	LONG-TERM
θ	$Oa-GFP_{\theta }$	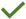	
	$Fm-GFP_{\theta }$	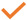	
	$As-GFP_{\theta }$		
α	$Oa-GFP_{\alpha }$	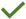	
	$Fm-GFP_{\alpha }$	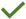	
	$As-GFP_{\alpha }$		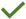
Lβ	$Oa-GFP_{L\beta }$	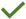	
	$Fm-GFP_{L\beta }$	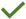	
	$As-GFP_{L\beta }$		
Hβ	$Oa-GFP_{H\beta }$		
	$Fm-GFP_{H\beta }$	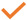	
	$As-GFP_{H\beta }$		
EDA	$SCR_{norm}$	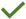	
	$SCL_{norm}$		

## Data Availability

The data that support the findings of this study are available from the corresponding authors upon reasonable request.
